# A Monstrosity

**Published:** 1887-06

**Authors:** J. Emmett Blackshear

**Affiliations:** Macon, Ga.


### A MONSTROSITY.*

BY J. EMMETT BLACKSHEAR, M. D., MACON GA.

On the 10th instant I was summoned to the house of Mrs. F., she being reported as threatened with abortion. She was a woman forty years of age, in good health, and the mother of four healthy children. She stated that she had passed the seventh month of pregnancy, and had been having labor-pains for about six hours. While I was preparing to make an examination a pain came on, and before I could get to her she exclaimed: "It is all over! thank God!"

The child proved to be a monster, resembling in external appearance more a frog than a human being. It was of the male sex and weighed three pounds. The head was flattened upon the top, the eyes and ears being near the upper border. There was no neck, the integuments of the chest coming out even with the chin. The right arm and hand were normal, except that the thumb was turned backwards. There was no forearm on the left side, the arm terminating at what should have been the elbow, with what seemed to be an effort at the formation of a hand, having only three fingers. The hips and lower extremities were very well formed, except that some of the toes were distorted. The spinal column was so broad as to give it the appearance of being double; dissection, however, proved this not to be the case.

I was assisted in the dissection by Drs. Fuller and Green. On examining the head we found that the frontal and parietal bones lay flat upon the base of the cranium, no cerebral substance whatever intervening. The occipital bone was entirely wanting, and in lieu thereof was what appeared to be the atlas, the anterior arch (perhaps) rising above the bones aforesaid. Underneath this arch a tuft of sanguinary fibrous tissue protruded, flowing down the back.

*Read before the Macon Medical Society.

The spinal cord, as well as the brain, was entirely wanting, the vertebræ being solid. The spinal column, together with what should have been the pelvic bones, the ribs, the clavical and the scalpula, were cartilaginous, ossification not having taken place. The bones, nerves and blood-vessels of the extremities were normal, except that the left arm was wanting, as before stated. I dissected out the sacrum with the sciatic nerve attached, which I dried and have here present.

The bronchi terminated in two rudimentary lungs, each about the size of a buck-shot or a little larger. The œsophagus terminated in the stomach, which, with the whole of the alimentary canal, except that portion of the large intestine which extended directly from the anus to and through the diaphragm on the left side, were contained in the left side of the thoracic cavity, crowding the heart into the right side. The heart was rudimentary, the divisions between the auricles and ventricals being very imperfect. It was attached to what appeared to be an effort at the formation of the aorta, which was, however, soon lost in the integuments of the spine. There was no connection whatever between the heart and lungs.

The liver was of immense size, occupying the whole of the abdominal cavity and extending above the diaphragm into the right side of the thoax. The kidneys, too, were much above the normal size.

Altogether it was one of the wildest freaks of nature with which I have ever met. The cause of such a departure from the laws of nature is a mystery that, in my opinion, admits of no satisfactory solution. The mother informed me that she had had no fright during the period of gestation, had not been unduly impressed in any way, had had no unnatural longings, and that nothing, to her knowledge, had occurred of a character to impress the fetus in utero.

